# The relationship between CALLY index and stroke in hypertensive patients: insights from NHANES

**DOI:** 10.3389/fnut.2025.1592641

**Published:** 2025-05-26

**Authors:** Xingfu Fan, Di Qing, Jin Zhao, Yang Luo, Xiaofang Li, Wenqin Tan, Shiping Liu

**Affiliations:** Department of General Medicine, Affiliated Hospital of North Sichuan Medical College, Nanchong, China

**Keywords:** Cally index, stroke, nutritional, inflammation, NHANES

## Abstract

**Background:**

At present, stroke ranks as the third leading cause of mortality, and hypertension is a major risk factor for stroke. Complementary assessment of inflammation level, immunity, and nutritional status is now possible using the newly developed C-reactive protein-albumin-lymphocyte (CALLY) index biomarker. One key concern in this study is that its correlation with the risk of stroke in individuals with hypertensiveness.

**Methods:**

In this study, we used cross-sectional analyses from the National Health and Nutrition Examination Survey (NHANES) database through 2003 to 2010. The CALLY index was calculated by albumin and lymphocytes divided by C-reactive protein (CRP). In order to further analysis, the CALLY index was log-transformed to increase data normality and lessen the impact of extreme values on the analytical findings. We investigated the odds ratios and confidence intervals of the ln CALLY index and its components in connection to stroke in people with hypertension. A weighted multivariable logistic regression model was carried out. Additionally, we used weighted restricted cubic splines (RCS) and subgroup analyses to further examine the association between the CALLY index and stroke prevalence in hypertensive individuals.

**Results:**

This study included 8,146 hypertensive participants, of whom 616 hypertensive participants had a stroke. In unadjusted modeling, we found a 39% reduction in the incidence of stroke in the hypertensive population in the highest ln CALLY quartile group (OR 0.61, 95% CI 0.46–0.82), and the negative association remained significant after adjustment for confounders. While ALB showed a robust protective impact in hypertensive people, with greater ALB levels linked to a decreased risk of stroke (OR 0.50, 95% CI 0.37–0.68), we also discovered a positive correlation between CRP and stroke risk (OR 1.13, 95% CI 1.04–1.22). A substantial correlation between the ln CALLY index and stroke risk in hypertensive individuals was also validated by subgroup analysis. The ln CALLY index and stroke risk in this sample also showed a strong linear negative connection, according to weighted restricted cubic spline (RCS) analysis.

**Conclusion:**

There is a significant negative association between the CALLY index and stroke risk in hypertensive patients in the U.S. adults. The CALLY index may be a potential indicator for early identification of individuals at higher risk of stroke in hypertensive patients and provide potential for clinical intervention.

## 1 Introduction

The Global Burden of Disease study states that stroke is the third leading cause of death worldwide, after ischemic heart disease and COVID-19 (1). The global prevalence of stroke has reached 100 million people, with a mortality rate of 87.4 per 100,000 people (1). Stroke predominantly results from an interruption in cerebral blood flow, causing ischemic and hypoxic conditions within the brain tissue (2). This condition is mainly categorized into two types: ischemic stroke and hemorrhagic stroke (2). Despite notable advancements in stroke management, various complications, including motor dysfunction, cognitive-linguistic disorders, swallowing difficulties, and anxiety-depression, persist (3). These complications significantly diminish the quality of life, increase psychological burden, and impose heavy economic costs on patients, families, and society. Therefore, promptly identifying high-risk groups and proactively managing relevant risk factors are crucial for the effective prevention of stroke.

Recent studies highlight hypertension as a significant contributor to stroke risk (4). Hypertensive patients are 9.7 times more likely to experience a stroke compared to individuals with normal blood pressure (5). Previous studies have shown that a persistent, long-lasting systemic inflammatory response is caused by hypertension. This is thought to be crucial for hypertensive individuals to experience a stroke (6, 7). Patients with hypertension have higher inflammatory markers. These chemical mediators create atherosclerosis, stimulate immune cells, and harm vascular endothelial cells, and all of them enhance the risk of stroke (8–10). Additionally, these inflammatory mediators can raise C-reactive protein (CRP) levels, which has been connected to a higher risk of stroke (11, 12). However, inflammation often comes with immune system dysfunction. Especially, hypertensive patients have higher levels of CD4^+^ and CD8^+^ T cells than those with normal blood pressure. These cells release inflammatory cytokines that invade the brain, triggering vascular and nervous inflammation, which in turn raises the risk of stroke (13, 14). Additionally, malnutrition is common among stroke survivors and can significantly worsen their prognosis (15). Studies have shown that serum albumin, the most abundant protein in circulation, affects atherosclerosis by influencing inflammation, platelet aggregation, and antioxidant activity (16). Consequently, inflammation, immune response, and nutritional factors play key roles in the development of stroke in individuals with hypertension.

A study conducted by Iida et al. (17) introduced the C-reactive protein-albumin-lymphocyte index (CALLY), a comprehensive biomarker that integrates CRP, albumin levels, and lymphocyte count to assess inflammation, immunological status, and nutritional condition (18). Prior investigations have revealed that the CALLY index is a potent indicator of negative cardiovascular consequences in individuals who have experienced ST-segment elevation myocardial infarction (19). Moreover, Wu et al. (20) observed a negative correlation between the CALLY index and metabolic syndrome. Further investigations have revealed significant associations between the CALLY index and various conditions, including sarcopenia, cancer, and chronic obstructive pulmonary disease (21–24), thereby validating its role as a reliable prognostic marker. Nevertheless, the association between the CALLY index and stroke risk in hypertensive populations remains to be clarified.

In order to fill this research gap, the present study delved into the relationship between the CALLY index and the incidence of stroke in patients with confirmed hypertension using cross-sectional analysis of NHANES data. This approach aims to offer a novel framework and theoretical foundation for the early prediction and prevention of stroke in hypertensive populations.

## 2 Materials and methods

### 2.1 Data sources and study participants

The National Health and Nutrition Examination Survey, or NHANES, is a significant survey conducted in the US to evaluate the general health of Americans through questionnaires, laboratory testing, and examinations. It collects information on health status, dietary habits, nutritional intake, and chronic diseases. Based on this, we utilized the NHANES dataset from 2003 to 2010 for cross-sectional analyses.

**Figure 1:**
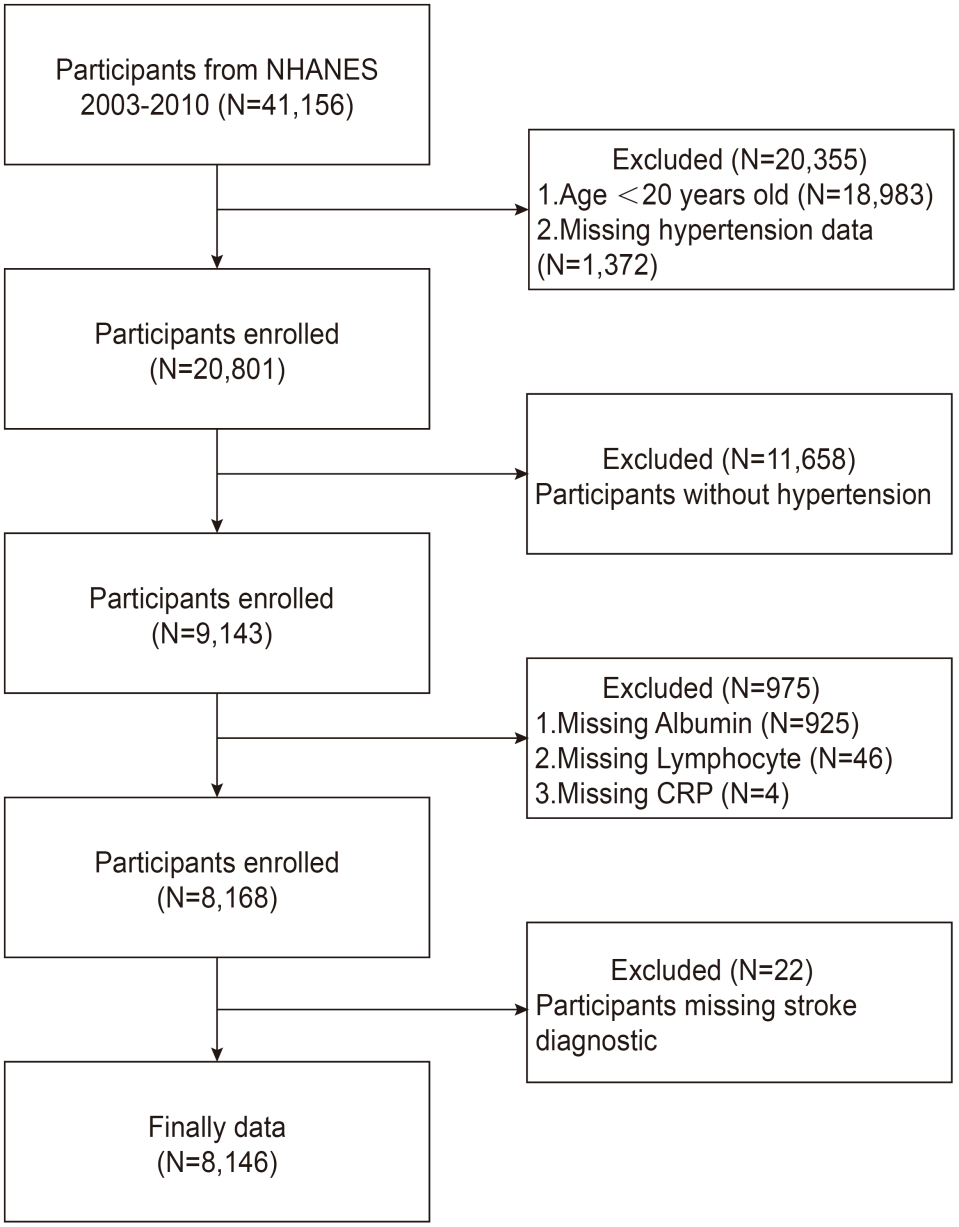
Flowchart of participant inclusion and exclusion in the current study.

From 2003 to 2010, we included a total of 41,156 participants from the NHANES database. To guarantee the quality and dependability of the study’s findings, strict exclusion criteria were applied based on previous studies (25–27). The exclusion criteria used in our study were as follows: (1) patients younger than 20 years or lacking hypertension data (*n* = 20,355); (2) patients without hypertensive disorders (*n* = 11,658); (3) patients with missing albumin, lymphocyte, or CRP data (*n* = 975); and (4) patients without stroke diagnostic information (*n* = 22). Ultimately, 8,146 hypertensive patients were included for further analysis, including 616 stroke survivors, as shown in Figure 1.

### 2.2 Calculation of the CALLY index

The serum albumin, lymphocyte and CRP blood indices for each cycle are measured by an automated blood tester. The calculation of the CALLY index is determined by the following formula (28, 29):


$$\text{CALLY index}=\frac{\text{Albumin}\;(g/L)\times \text{Lymphocyte}\;(1,000\;\text{cells}/\mathrm{\mu L})}{\mathrm{CRP}\;(\mathrm{mg}/\mathrm{dL})}$$

To improve the normality of the data and reduce the influence of extreme values on the analytical results, the CALLY index (original variable) was log-transformed prior to analysis. Specifically, all values of the CALLY index were converted to their natural logarithmic form (ln CALLY) to better satisfy the assumptions of linear regression and enhance the robustness of the model. This transformation facilitated a distribution closer to normality and improved the linearity of the association between the CALLY index and stroke risk, thereby strengthening the reliability and interpretability of the results.

### 2.3 Diagnosis of hypertension

Hypertension was defined based on prior studies (30, 31), as follows: (1) a mean systolic blood pressure of at least 140 mmHg; (2) a mean diastolic blood pressure of at least 90 mmHg; (3) answer “Yes” to the following questionnaire questions: “Has a doctor or health professional ever told you that you have hypertension, also known as high blood pressure (HBP)?” or “Are you currently taking prescribed medication for high blood pressure?”

### 2.4 Evaluation of stroke

Stroke status of participants was determined based on questionnaire on medical history from NHANES. In the questionnaire, professionals asked, “Has a doctor or other medical professional ever told you about a stroke?” People who answered “yes” to this question were considered to have had a stroke.

### 2.5 Definition of covariates

The covariates considered in this study include age, sex, ethnic origin (including Mexican American, non-Hispanic White, non-Hispanic Black, other Hispanic, and other racial categories), level of education (classified as less than beneath high school, high school, and high school diploma or higher), marriage (married, unmarried), body mass index (BMI, in kg/m^2^) (≥30, 25–30, <25), drinking consumption (have you consumed more than 12 alcoholic beverages in the past year), smoking (never smoker, ever smoker, current smoker), and questionnaire with history of diabetes, heart failure (HF), and coronary artery heart disease (CHD). Moreover, fasting blood glucose (FBG) levels, triglycerides (TG) levels, total cholesterol (TC) levels, high-density lipoprotein cholesterol (HDL-C) levels, low-density lipoprotein cholesterol (LDL-C) levels, and waist circumference (WC) were also recorded.

### 2.6 Statistical analysis

Sample weights were calculated using the NHANES-recommended Full Sample 2-Year MEC Exam Weight (WTMEC2YR), divided by 4, with clustering and stratification incorporated into all analyses. Whereas classification variables are displayed as frequencies (*n*, %), continuous variables are displayed as mean ± standard deviation. Differences in participant characteristics across CALLY index quartiles were assessed using one-way analysis of variance (ANOVA) for continuous variables and Pearson’s chi-square test for categorical variables. The relationship between the CALLY index and stroke risk in hypertensive individuals was examined using weighted multivariate logistic regression models. The crude model was unadjusted, while Model 1 adjusted for age, race, sex, education, and marriage. Model 2 further adjusted for smoking, drinking consumption, diabetes, HF, FBG, TC, HDL-C, TG, BMI, and WC, in addition to the covariates from Model 1. Potential non-linear associations were examined through restricted cubic spline (RCS) analysis. Subgroup and interaction analyses were performed to assess heterogeneity across different strata. Missing data were imputed using random forest interpolation. R Studio was used to conduct the statistical analysis, and a two-tailed *p*-value threshold of less than 0.05 was used to assess significance.

### 2.7 Statement of morals

The National Center for Health Statistics’ (NCHS) Ethics examination Board approved the NHANES datasets used in this study after they undergone a thorough examination. Informed consent was obtained in writing from all participants prior to their involvement. Given that the study relied on the analysis of publicly available datasets, all participant data were anonymized and de-identified, thereby exempting the research from the need for ethical approval from local institutions.

## 3 Results

### 3.1 Baseline analysis

Data from 8,146 participants were analyzed, with baseline characteristics shown in Table 1. The average age of the cohort was 56.98 ± 15.40 years, with 48.96% male and 51.04% female participants, 73.93% of whom identified as non-Hispanic White. Among the participants, 616 (6.10%) had a stroke diagnosis. Those in the top quarter of the ln CALLY index were more probable to be married, overweight, male, and well-educated than those in the lowest group. Regarding lifestyle factors, the high ln CALLY group had a higher proportion of never-smokers and alcohol consumers. With respect to comorbidities, the prevalence of diabetes and heart failure was lower in the highest ln CALLY index quartile. In terms of blood biomarkers, this group showed elevated fasting blood glucose levels, C-reactive protein, total cholesterol, in contrast triglyceride levels were reduced. Additionally, this group had higher albumin concentrations, lymphocyte counts, and HDL cholesterol levels.

**Table 1:** Weighted patient demographics and baseline characteristics of participants according to ln CALLY index.

Variable	Total	Q1 (<1.82)	Q2 (1.82–2.81)	Q3 (2.81–3.74)	Q4 (>3.74)	*p*-value
	*N* = 8,146	*N* = 2,036	*N* = 2,036	*N* = 2,005	*N* = 2,069	
Age (years)	56.98 ± 15.40	56.56 ± 15.63	57.61 ± 14.95	57.48 ± 14.98	56.30 ± 15.94	0.010
Sex, (%)						<0.001
Male	4,043 (48.96%)	827 (36.83%)	974 (47.54%)	1,078 (52.98%)	1,164 (56.54%)	
Female	4,103 (51.04%)	1,209 (63.17%)	1,062 (52.46%)	927 (47.02%)	905 (43.46%)	
Race (%)						<0.001
Mexican American	1,245 (5.35%)	329 (5.82%)	320 (5.32%)	318 (5.43%)	278 (4.90%)	
Other Hispanic	493 (3.04)	134 (3.46%)	112 (2.67%)	123 (2.81%)	124 (3.24%)	
Non-Hispanic White	4,245 (73.93%)	954 (69.55%)	1,076 (74.87%)	1,096 (76.60%)	1,119 (74.25%)	
Non-Hispanic Black	1,858 (12.86%)	567 (17.34%)	462 (13.37%)	390 (10.37%)	439 (11.02%)	
Other race	305 (4.82%)	52 (3.83%)	66 (3.77%)	78 (4.79%)	109 (6.59%)	
Education (%)						<0.001
<High school	2,673 (21.81%)	734 (25.05%)	681 (22.47%)	662 (20.89%)	596 (19.41%)	
High school	2,045 (27.02%)	488 (26.84%)	548 (28.89%)	497 (27.51%)	512 (25.05%)	
>High school	3,428 (51.17%)	814 (48.11%)	807 (48.64%)	846 (51.60%)	961 (55.54%)	
Marriage, (%)						<0.001
Married	4,479 (60.55%)	1,017 (56.03%)	1,146 (61.14%)	1,139 (62.20%)	1,177 (62.24%)	
Non-married	3,667 (39.45%)	1,019 (43.97%)	890 (38.86%)	866 (37.80%)	892 (37.76%)	
Drinking (%)						<0.001
Yes	5,585 (71.58%)	1,309 (65.87%)	1,355 (68.12%)	1,416 (74.11%)	1,505 (76.99%)	
No	2,561 (28.42%)	727 (34.13%)	681 (31.88%)	589 (25.89%)	564 (23.01%)	
Smoking status, (%)						<0.001
Current smoker	1,182 (14.26%)	279 (12.65%)	255 (13.06%)	309 (14.82%)	339 (16.13%)	
Former smoker	3,874 (48.33%)	1,160 (60.14%)	1,098 (54.23%)	893 (44.85%)	723 (36.63%)	
Never smoker	3,090 (37.41%)	597 (27.21%)	683 (32.71%)	803 (40.33%)	1,007 (47.24%)	
Stroke, (%)						<0.001
Yes	616 (6.10%)	191 (8.25%)	154 (5.89%)	141 (5.44%)	130 (5.10%)	
No	7,530 (93.90%)	1,845 (91.75%)	1,882 (94.11%)	1,864 (94.56%)	1,939 (94.90%)	
Diabetes, (%)						<0.001
Yes	1,664 (15.92%)	473 (19.84%)	425 (17.13%)	388 (13.89%)	378 (13.50%)	
No	6,482 (84.08%)	1,563 (80.16%)	1,611 (82.87%)	1,617 (86.11%)	1,691 (86.50%)	
Heart failure, (%)						<0.001
Yes	519 (4.92%)	186 (7.51%)	127 (4.80%)	116 (4.57%)	90 (3.21%)	
No	7,627 (95.08%)	1,850 (92.49%)	1,909 (95.20%)	1,889 (95.43%)	1,979 (96.79%)	
Coronary artery heart disease (%)						0.871
Yes	654 (7.16%)	164 (7.54%)	152 (6.95%)	181 (7.26%)	157 (6.95%)	
No	7,492 (92.84%)	1,872 (92.46%)	1,884 (93.05%)	1,824 (92.74%)	1,912 (93.05%)	
BMI (kg/m^2^)						<0.001
<25	1,646 (20.39%)	284 (12.77%)	330 (15.47%)	406 (19.86%)	626 (31.47%)	
≥25, <30	2,697 (32.90%)	522 (23.95%)	647 (31.61%)	709 (35.18%)	819 (39.31%)	
≥30	3,803 (46.71%)	1,230 (63.28%)	1,059 (52.92%)	890 (44.96%)	624 (29.22%)	
Albumin (g/L)	4.21 ± 0.34	4.02 ± 0.34	4.18 ± 0.35	4.26 ± 0.30	4.34 ± 0.29	<0.001
FPG (mg/dL)	114.96 ± 26.13	116.37 ± 31.75	116.05 ± 28.83	115.03 ± 24.11	112.79 ± 19.24	<0.001
CRP (mg/dL)	0.49 ± 0.86	1.31 ± 1.46	0.46 ± 0.36	0.24 ± 0.17	0.09 ± 0.08	<0.001
Lymphocyte (1,000 cells/dL)	1.34 ± 1.09	0.77 ± 0.58	1.12 ± 0.83	1.46 ± 0.96	1.89 ± 1.40	<0.001
TC (mg/dL)	201.19 ± 42.53	201.08 ± 43.61	202.04 ± 43.10	204.12 ± 42.52	197.85 ± 40.89	<0.001
HDL-C (mg/dL)	52.40 ± 16.48	50.44 ± 15.98	50.50 ± 14.96	52.42 ± 16.11	55.68 ± 17.92	<0.001
TG (mg/dL)	154.27 ± 76.34	157.20 ± 81.37	157.43 ± 83.23	159.38 ± 78.91	144.39 ± 60.89	<0.001
LDL-C (mg/dL)	114.84 ± 24.59	115.10 ± 25.48	115.34 ± 25.27	114.88 ± 23.43	114.15 ± 24.24	0.426
Waist circumference (cm)	103.97 ± 15.51	108.84 ± 16.03	106.64 ± 15.40	103.36 ± 14.50	98.19 ± 14.07	<0.001

Q, quartile; FPG, fasting plasma glucose; CRP, C-reactive protein; TC, total cholesterol; HDL-C, high-density lipoprotein cholesterol; TG, triglyceride; LDL-C, low-density lipoprotein cholesterol; BMI, body mass index.

### 3.2 The association between the ln CALLY index and its components with stroke incidence in individuals with hypertension

**Figure 2:**
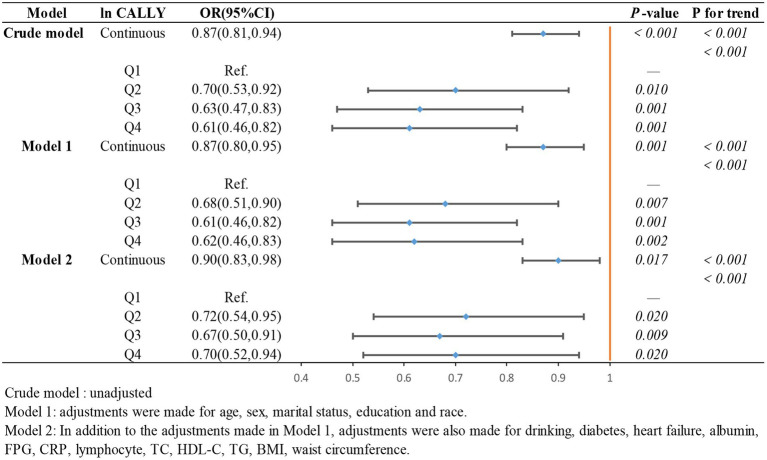
The association between Ln CALLY index and stroke occurrence in hypertensive participants.

To further investigate the relationship between the ln CALLY index and its components with stroke incidence in individuals with hypertension and to present the results more intuitively, we constructed three logistic regression models to examine the independent effects of the ln CALLY index and its components on stroke incidence. When the ln CALLY index was treated as a continuous variable (see Figure 2), we observed a significant negative correlation between stroke risk and the ln CALLY index in hypertensive individuals (OR 0.87, 95% CI 0.81–0.94). After categorizing the ln CALLY values into four quartiles, with the first quartile as the reference group, in the unadjusted model, the highest quartile of ln CALLY showed a 39% reduction in stroke incidence compared to the reference group (OR 0.61, 95% CI 0.46–0.82). In Model 2, after adjusting for demographic characteristics, blood parameters, and comorbidities, the highest quartile of ln CALLY remained negatively associated with stroke incidence, with a 30% reduction in stroke risk for each 1-unit increase in ln CALLY.

**Figure 3:**
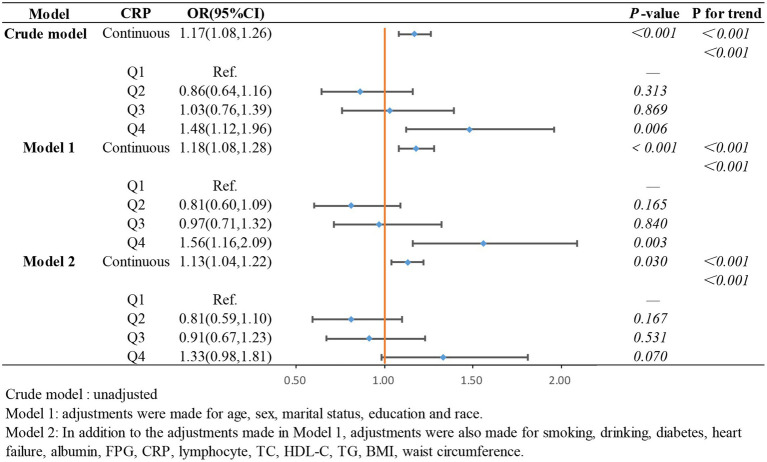
The association between CRP and stroke occurrence in hypertensive participants.

**Figure 4:**
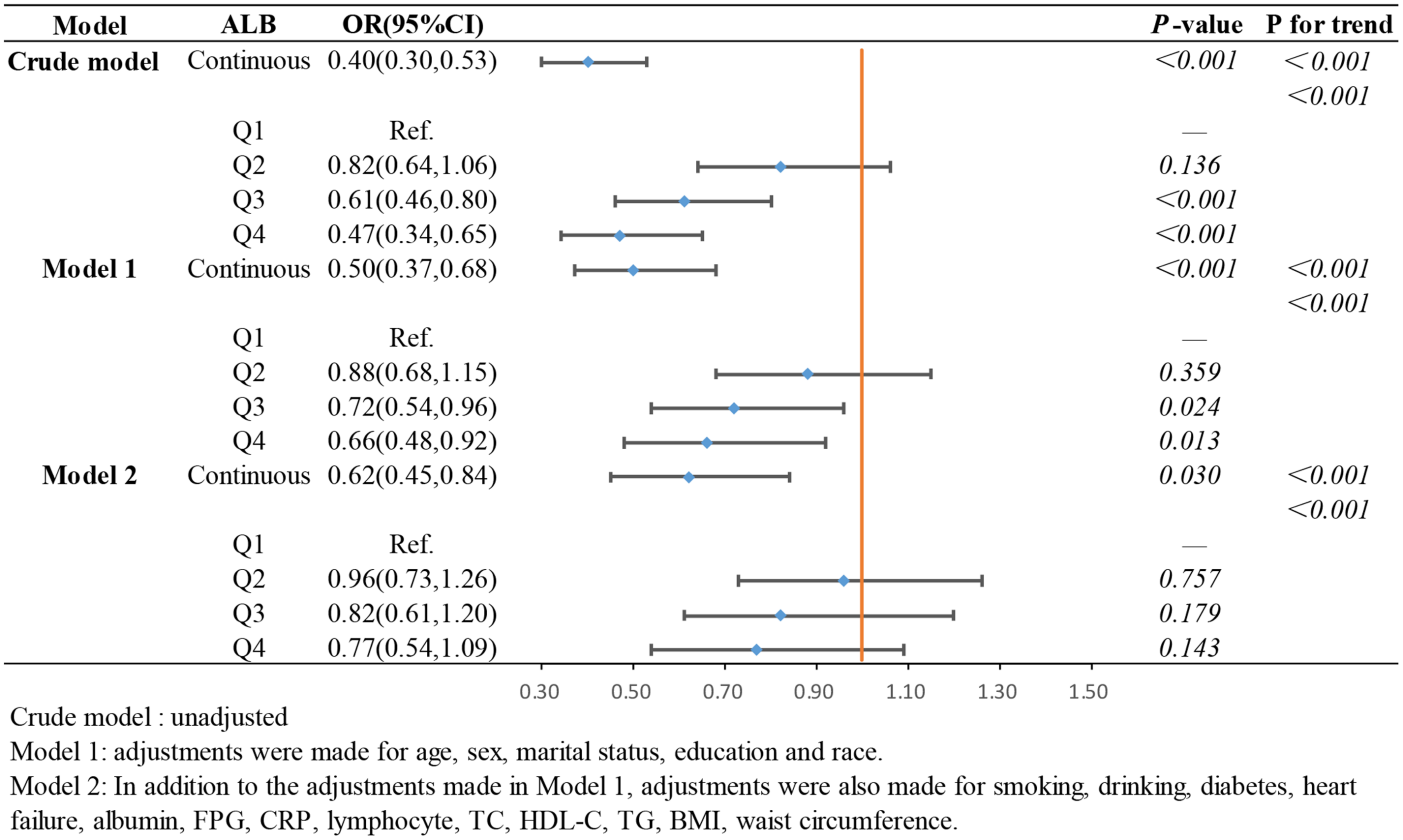
The association between albumin and stroke occurrence in hypertensive participants.

**Figure 5:**
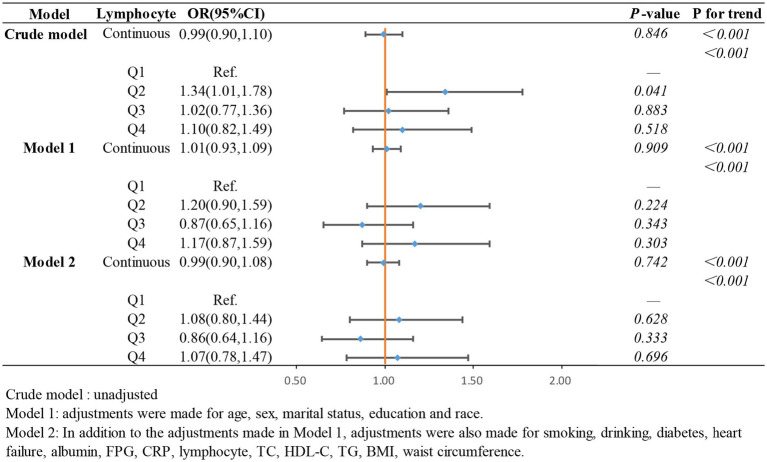
The association between lymphocyte count and stroke occurrence in hypertensive participants.

In the weighted logistic regression analysis further exploring the relationship between the components of ln CALLY and stroke risk in hypertensive individuals, Figure 3 shows that CRP is positively correlated with stroke risk in Model 2 (OR 1.13, 95% CI 1.04–1.22). Additionally, we found that in Model 2, the OR for the continuous variable decreased from 1.18 to 1.13 compared to the unadjusted model, indicating a slight attenuation of this relationship after adjusting for confounders. In contrast, Figure 4 shows that ALB exerts a strong protective effect in hypertensive individuals, with higher ALB levels associated with lower stroke risk (OR 0.50, 95% CI 0.37–0.68). Figure 5 demonstrates that lymphocyte count, as a continuous variable, is not significantly associated with stroke risk in hypertensive individuals. Trend analysis across all models (*p* trend <0.001) consistently reveals a significant upward trend.

**Figure 6:**
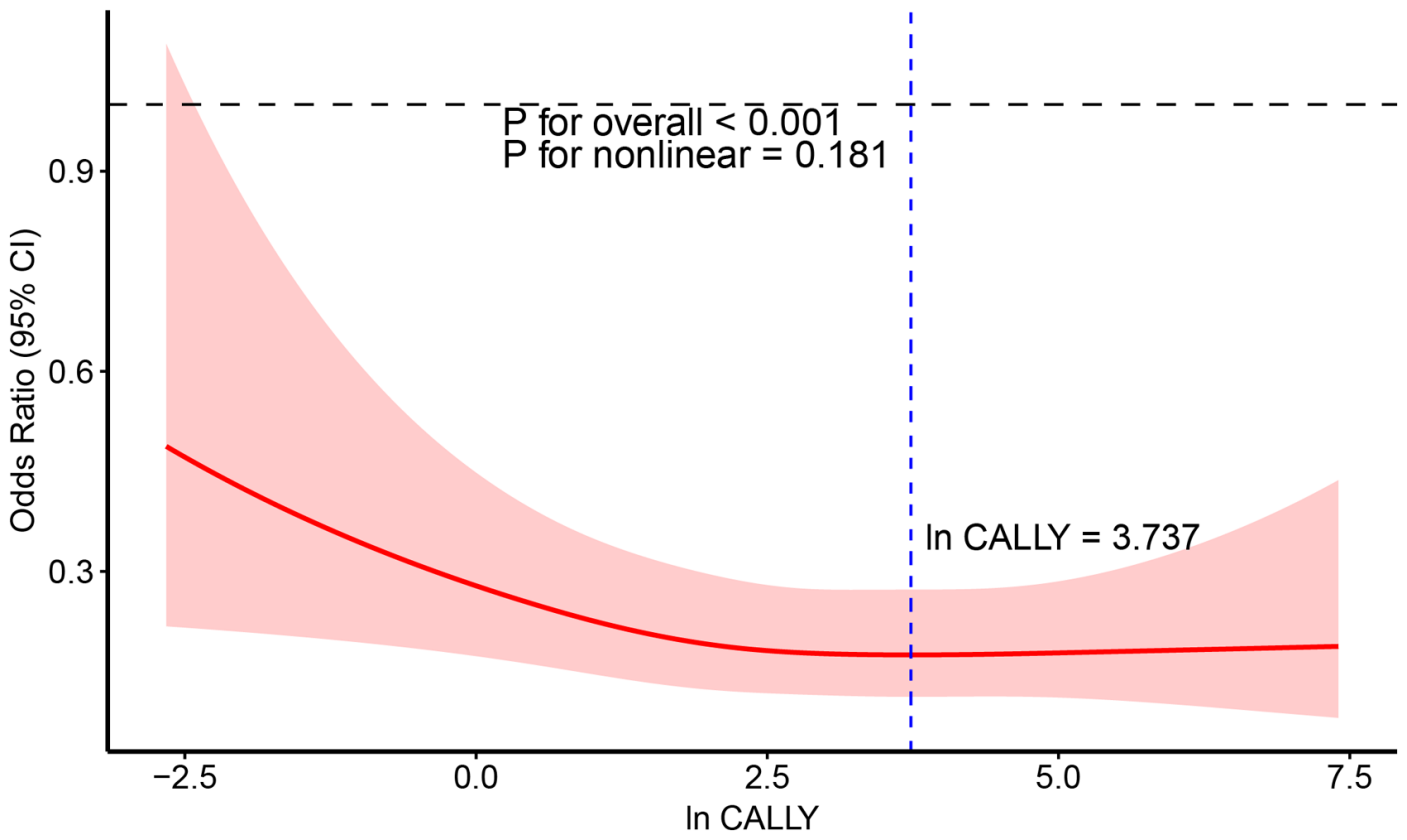
RCS analysis of the relationship between Ln CALLY index and stroke occurrence in hypertensive participants.

Furthermore, the weighted restricted cubic spline (RCS) analysis was used to visualize the relationship between the ln CALLY index and stroke incidence in hypertensive individuals, as shown in Figure 6. The RCS curve analysis indicates a significant linear negative correlation between ln CALLY and stroke incidence (*p* < 0.0003). As ln CALLY increases, stroke incidence in hypertensive individuals decreases significantly. However, when the ln CALLY value reaches 3.737, further increases in ln CALLY lead to a plateau in the negative correlation.

### 3.3 Subgroup analyses

**Figure 7:**
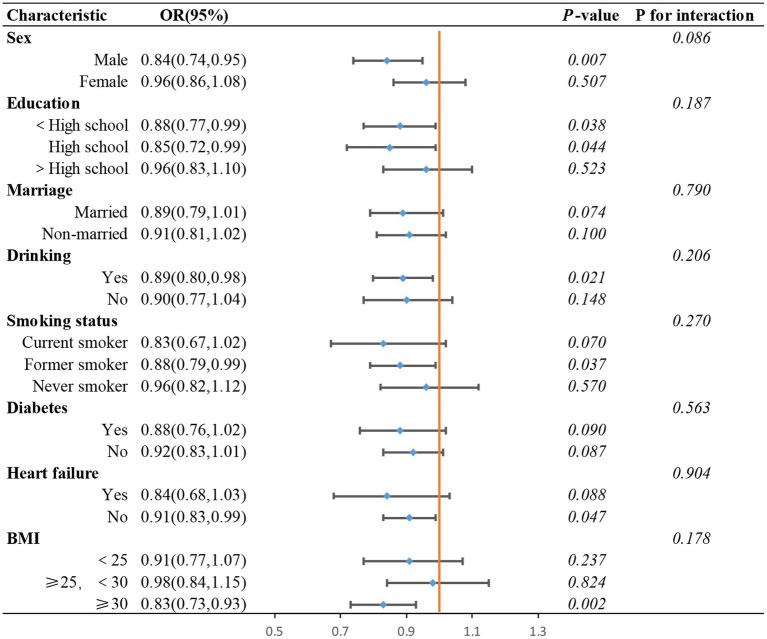
Subgroups analysis of the association between ln CALLY index and stroke in hypertensive populations pectoris.

To assess the stability of the relationship between the ln CALLY index and stroke risk in hypertensive individuals across different subgroups, we conducted subgroup analyses and tested for interaction effects. In the subgroup analysis using ln CALLY as a continuous variable (see Figure 7), we found that individuals with BMI ≥30 showed a significantly lower stroke risk with higher ln CALLY values (OR = 0.83, 95% CI: 0.73–0.93, *p* = 0.002), suggesting that a higher ln CALLY index may have a stronger protective effect in the high BMI population. Additionally, the negative correlation between ln CALLY and stroke risk was more pronounced in males (OR = 0.84, 95% CI: 0.74–0.95, *p* = 0.007), indicating that an increase in ln CALLY may be associated with a reduction in stroke risk in men. In contrast, no significant relationship was observed between ln CALLY and stroke risk in females (*p* = 0.507).

**Figure 8:**
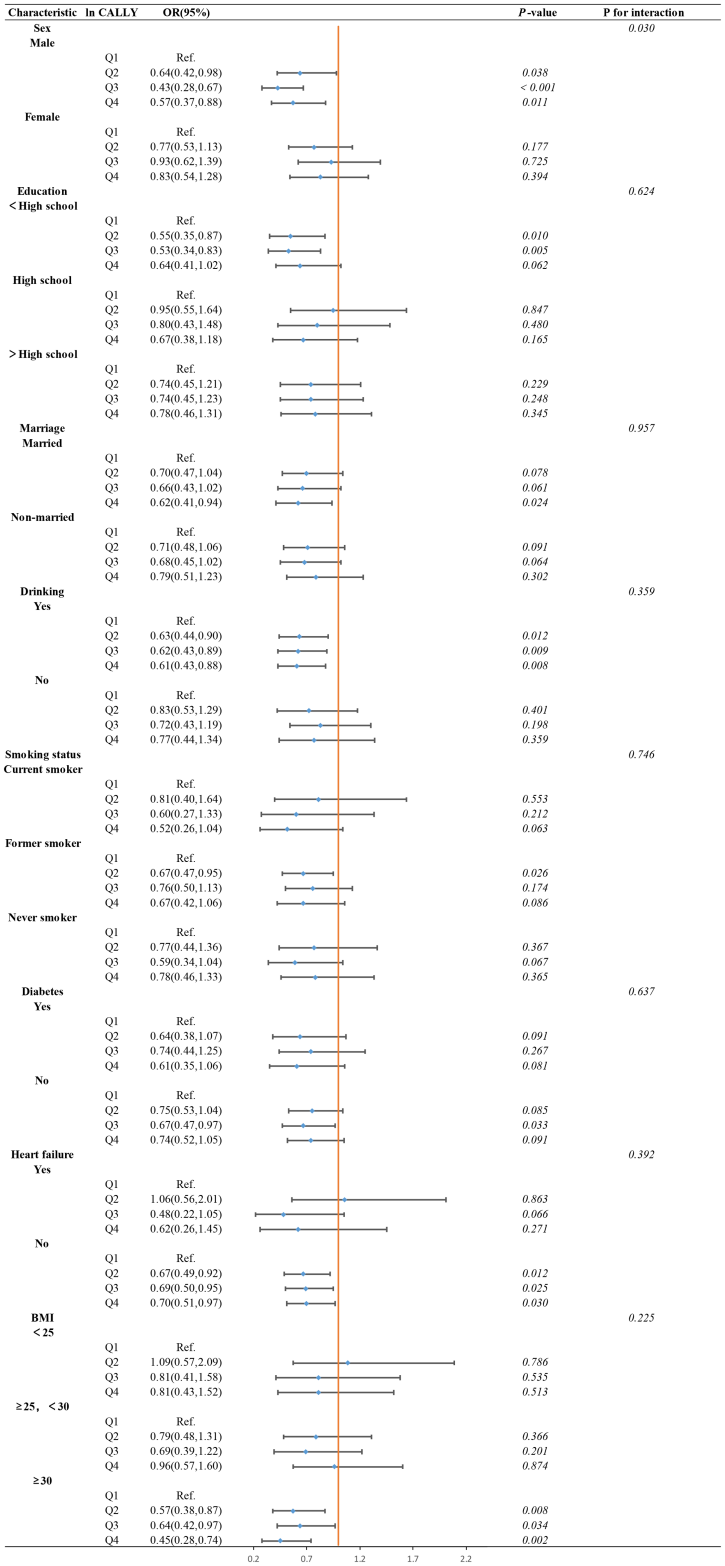
Subgroups analysis of the association between ln CALLY index quartiles and stroke in hypertensive populations pectoris.

However, in the subgroup analysis using ln CALLY quartiles (see Figure 8), we found that the interaction between gender and ln CALLY quartiles significantly influenced stroke risk. Specifically, males in the Q2, Q3, and Q4 groups had a significantly lower stroke risk compared to the reference group (Q1), with *p*-values of 0.038, <0.001, and 0.011, respectively. In contrast, no statistically significant differences were found between the quartiles and the reference group in females (*p*-values of 0.177, 0.725, and 0.394). These results suggest that the association between ln CALLY and stroke differs by gender. Furthermore, in the analysis of ln CALLY index quartiles, we observed a stronger negative correlation between the fourth quartile and the first quartile in individuals who were married, consumed alcohol, had no diabetes, had no heart failure, and had BMI ≥30.

By comparing the subgroup analyses of ln CALLY index as both a continuous and categorical variable, we identified an interesting phenomenon. The interaction analysis of the continuous variable subgroup showed no significant interaction effects across subgroups (*p* for interaction >0.05). However, the interaction effects in the categorical variable subgroup analysis revealed a statistically significant gender interaction (*p* for interaction <0.05), with no significant interactions observed in other subgroups (*p* for interaction >0.05). This phenomenon may be due to the clearer group distinctions provided by the categorical variable (quartiles), which helps to observe effect variations within specific groups, making the gender effect more prominent. In contrast, in the continuous variable analysis, the relationship between gender and the ln CALLY index was treated as linear, which may not capture the complex interaction between gender and the ln CALLY index.

## 4 Discussion

Our study performed a cross-sectional analysis of 8,146 adult hypertensive patients to examine the association of the ln CALLY index with stroke prevalence. The results showed that among people with hypertension, the incidence of stroke decreased by 39% for every unit increase in the ln CALLY index. After limiting for potential confounders, this inverse connection persisted, suggesting that the CALLY index may serve as a protective factor in predicting the risk of stroke in this population. We further completed subgroup analyses and interaction tests, which showed a more documented relationship. In addition, significant gender differences were found in the subgroup analyses, with males being more affected.

The CALLY Index is a combined measure that reflects inflammation, immune function, and nutritional status. Hypertension induces systemic chronic inflammation through the classical inflammasome activation pathway (32). Long-term hypertension leads to vascular sclerosis, endothelial injury, and necrosis (33), which release damage-associated molecular patterns (DAMPs). These molecules further activate the NLRP3 inflammasome, promoting the release of inflammatory factors, thereby exacerbating neurovascular inflammation and promoting the development of atherosclerosis (34, 35). Additionally, upon binding to Toll-like receptors, DAMPs promote the release of pro-inflammatory cytokines such as IL-1β (36, 37). The release of these inflammatory factors increases the body’s inflammatory response, which in turn raises the risk of stroke in individuals with hypertension (11, 38). CRP, a well-established biomarker of inflammation (39), has been shown to predict cardiovascular events in a prospective study involving 27,939 American women, with a 30-year follow-up period (40). A Mendelian randomization study highlighted a clear causal relationship between CRP and stroke (41). Furthermore, a randomized double-blind trial demonstrated that individuals with elevated CRP levels had a 4.7-fold increased risk of recurrent stroke compared to those without elevated CRP (42). These findings suggest that elevated CRP promotes stroke occurrence, aligning with our study’s observation of an association between CRP and stroke risk in individuals with hypertension.

During the activation of inflammation, the immune system plays a critical role. DAMPs bind to Toll-like receptors, triggering the innate immune response and further activating T lymphocytes and B lymphocytes (43). T lymphocytes secrete pro-inflammatory cytokines that exacerbate vascular inflammation and cerebral tissue damage. On the other hand, B lymphocytes, produce autoantibodies that amplify immune responses and inflammation, further elevating stroke risk (44). Tregs (regulatory T lymphocytes) release anti-inflammatory cytokines, including IL-10 and TGF-β, reducing inflammation, minimizing brain tissue damage, and enhancing functional recovery. Tregs also inhibit immune responses by binding to dendritic cells through CTLA-4 receptors, suppressing dendritic cell activation, thereby suppressing immune responses and mitigating inflammation, which reduces stroke risk (45, 46). As indicated by a bidirectional Mendelian randomization study, both B lymphocytes and T lymphocytes have bidirectional effects on stroke risk (47). In our study, no clear positive or negative correlation was found between lymphocyte count and stroke. Further research is needed to explore and validate this potential association.

Nutrition is essential for cardiovascular health, and serum albumin, as an important nutritional marker, exhibits anti-inflammatory and antioxidant functions (48). First, albumin contains abundant thiol groups, acting as an antioxidant to scavenge reactive oxygen and nitrogen species (49). Second, low serum albumin leads to impaired fibrinolysis and reduced platelet aggregation, promoting the formation of atherosclerotic plaques or blood clots, thus increasing the risk of stroke (50). Finally, low serum albumin levels decrease red blood cell deformability, increasing blood viscosity and further elevating stroke risk (51). A study from the NHANES database involving 17,303 participants identified a negative correlation between serum albumin levels and stroke risk (15). Additionally, a cohort study from Japan found that hypoalbuminemia is associated with an increased risk of stroke (52). Our study also explored the relationship between albumin (ALB) levels and stroke risk in individuals with hypertension, and similarly demonstrated a negative correlation between serum albumin levels and stroke risk.

The CHA_2_DS_2_-VASc score has become a widely accepted tool for the clinical assessment and prediction of stroke risk. A prospective study involving 1,494 patients demonstrated that the CHA_2_DS_2_-VASc score offers superior clinical utility in forecasting acute ischemic stroke (53). Furthermore, a study of 62,227 patients noted that the CHA_2_DS_2_-VASc score independently predicted the occurrence of acute ischemic stroke (54). This scoring system incorporates clinical factors such as age, sex, and medical history, allowing for real-time assessment that is both cost effective and straightforward to implement. However, it primarily relies on long-term clinical characteristics rather than reflecting the patient’s current physiological state. This limits its sensitivity in younger populations and its ability to detect subtle short-term clinical fluctuations. The Framingham score is another tool used for stroke risk evaluation (55), and a study tracking 27,748 stroke-free individuals over 5.6 years revealed a positive correlation between higher Framingham scores and increased stroke risk (56). While the Framingham score is simple, affordable, and effective for long-term risk assessment, it also has notable limitations in capturing dynamic clinical conditions and assessing short-term stroke risk. In contrast, the CALLY index provides real-time physiological status through hematological markers, evaluating immune, nutritional, and inflammatory status from multiple dimensions, offering a high degree of objectivity.

Subgroup analyses in this study further indicated a more pronounced correlation of the CALLY index with the risk of stroke in hypertensive men (*p* for interaction = 0.0304). A meta-analysis of 59 studies from 19 countries across five continents found that stroke incidence is 33% higher in men compared to women (57). This difference may be attributed to the effects of sex hormones, which modulate cerebrovascular reactivity. Specifically, estrogen enhances endothelial nitric oxide synthase (eNOS) activity, increasing nitric oxide (NO) production, promoting vasodilation, improving vascular compliance, reducing arterial blood pressure, and minimizing endothelial cell damage, which collectively reduces stroke risk (58). Furthermore, estrogen inhibits the onset of atherosclerosis by suppressing the proliferation of vascular smooth muscle cells, reducing lipoprotein(a) accumulation, and decreasing the adhesion and differentiation of monocytes, thereby mitigating vascular wall damage and inflammatory responses (59). Additionally, the smaller size of women’s arteries and hearts may result in less vascular damage under similar hemodynamic conditions, contributing to a lower incidence of stroke in women (60, 61).

The CALLY index, as a reliable biomarker, offers clinicians a preliminary means to assess stroke risk in hypertensive patients. In comparison to traditional risk assessment methods, the CALLY index provides superior ease of use and real-time applicability, offering robust scientific support for early intervention and treatment. Research indicates that fluctuations in the CALLY index are closely linked to patients’ immune, inflammatory, and nutritional status, thereby justifying the adjustment of treatment strategies based on CALLY index levels, particularly in the domains of inflammation, immunity, and nutrition therapy. Our findings advocate for the implementation of a risk stratification approach to hypertension management in clinical practice. Evidence suggests that patients with a low CALLY index face a significantly heightened stroke risk. Consequently, for hypertensive patients exhibiting low CALLY index levels, proactive early interventions, including routine monitoring and optimal blood pressure control, should be prioritized, alongside personalized treatment strategies. This approach facilitates early identification of high-risk individuals and ensures timely, targeted interventions to reduce stroke incidence.

A key strength of this study is the use of the NHANES database, which ensures a nationally representative sample and strengthens the generalizability of the findings. In addition, we introduced the CALLY index as a novel, non-invasive biomarker that could improve stroke risk assessment in hypertensive individuals. However, some limitations of our study have to be recognized. First, the precision of the data may be impacted by the NHANES design, which permits the gathering of some measurements only at a single time point. Additionally, excluding people with insufficient information may result in selection prejudice. Second, as this study relies on the NHANES database, it is not possible to accurately determine whether participants with chronic diseases were also experiencing acute infections. Acute infections can cause an elevation in CRP levels, potentially compromising the stability of the CALLY index and its ability to predict outcomes. This is a limitation of our study. Finally, we cannot conclude causal links because of the retrospective and cross-sectional form of this study, which leaves our observations vulnerable to inherent biases. Notwithstanding these drawbacks, the results have clinical significance. Our research indicates a preliminary inverse relationship between the CALLY index and the risk of stroke in hypertensive populations, larger, multi-center clinical trials are required to validate its precision and wider application.

## 5 Conclusion

Our analysis suggests that in U.S. adults, the CALLY index is negatively correlated with stroke risk in hypertensive patients. The CALLY index may provide additional value in identifying individuals at higher stroke risk among hypertensive populations. Additional prospective studies are required to corroborate our findings.

## Data Availability

The original contributions presented in the study are included in the article/supplementary material, further inquiries can be directed to the corresponding author.
